# Supplementary material for: Causal interplay between lactose intolerance and gut microbiota: a combined bidirectional Mendelian randomization and in vivo validation study
Source: Front Nutr. 2026 Jun 1;13:1803337. doi: 10.3389/fnut.2026.1803337 (PMC13265576; doi:10.3389/fnut.2026.1803337)
Supplement: Supplementary file 1 [file Data_Sheet_1.zip › supplementary materials/Forward/leave one out cross validation/ebi-a-GCST90027468.finngen_R12_E4_LACTONAS.pdf]

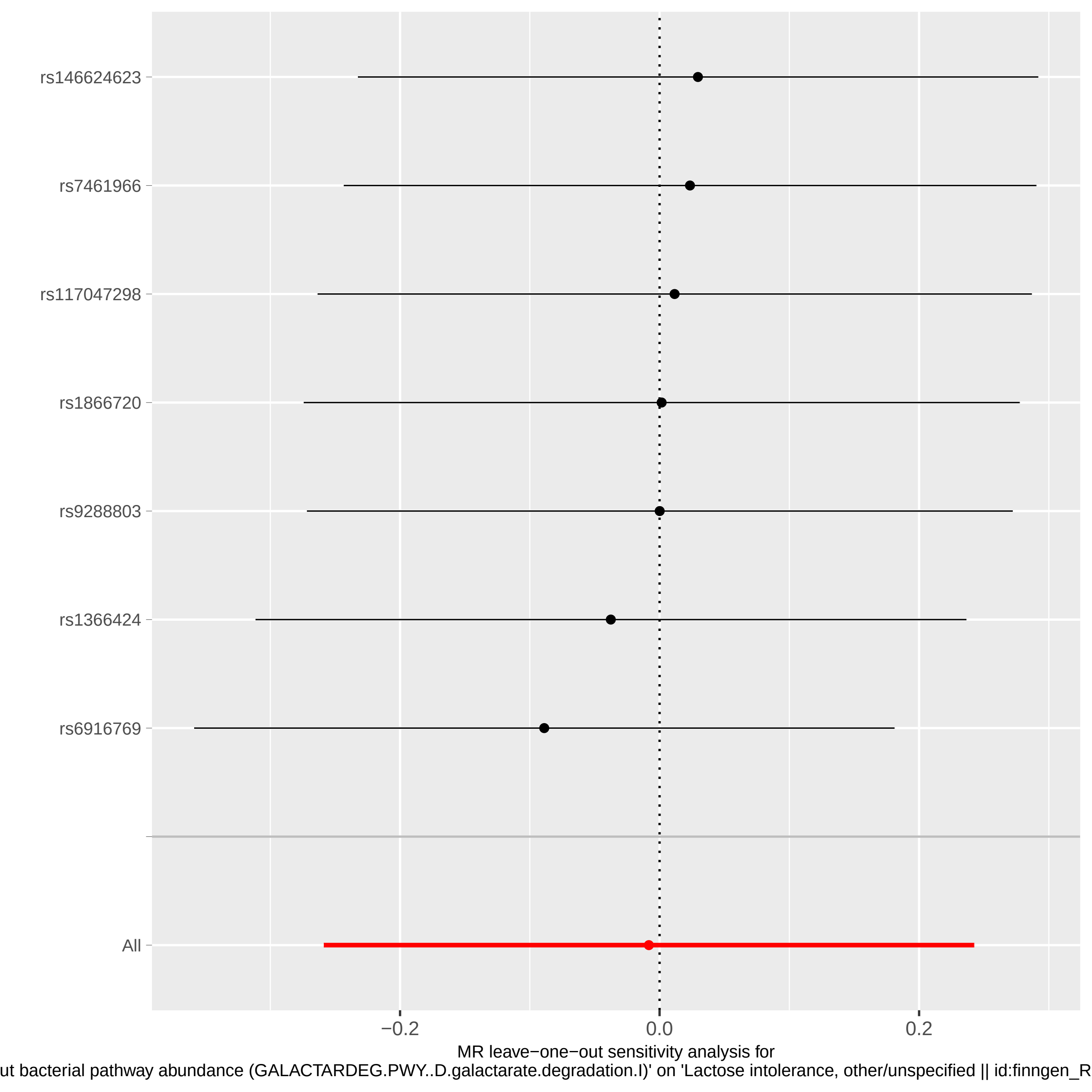

rs146624623
rs7461966
rs117047298
rs1866720
rs9288803
rs1366424
rs6916769
All
−0.2
0.0
0.2
MR leave−one−out sensitivity analysis for
ut bacterial pathway abundance (GALACTARDEG.PWY..D.galactarate.degradation.I)' on 'Lactose intolerance, other/unspecified || id:finngen_R
